# Protectin DX alleviates lumbar radicular pain in rats by suppressing RIP3/MLKL-associated necroinflammation through restoration of autophagic function

**DOI:** 10.3389/fphar.2026.1957244

**Published:** 2026-09-23

**Authors:** Qing-xiang Zhao, Xiao-juan Wu, Qing Yao, Yong Gao

**Affiliations:** 1 Department of Pain Management, Shandong Medical and Pharmaceutical University Hospital, Binzhou, Shandong Province, China; 2 College of Public Health, Shandong Second Medical University, Weifang, Shandong Province, China

**Keywords:** protectin DX, lumbar radicular pain, necroptosis, necroinflammation, autophagy, adenosine monophosphate-activated protein kinase, specialized pro-resolving lipid mediators

## Abstract

**Background:**

Lumbar radicular pain (LRP) is a prevalent neuropathic pain disorder driven by nucleus pulposus (NP)-induced inflammation. Protectin DX (PDX), a novel specialized pro-resolving lipid mediator (SPM), exerts pro-resolving and analgesic effects in LRP. However, the underlying molecular mechanisms remain unclear. Emerging evidence suggests that crosstalk between necroptosis and autophagy coordinately regulates cell fate and inflammatory responses. This study therefore investigated whether this crosstalk mediates the therapeutic effects of PDX in LRP.

**Methods:**

Rat models of LRP were established by transplanting autologous NP onto the L5 dorsal root ganglion. The rats received intrathecal injections of PDX (10, 100, or 200 ng), GSK'872 (a receptor-interacting protein kinase 3 (RIP3) inhibitor), chloroquine (CQ; an autophagy inhibitor), or Compound C (an adenosine monophosphate-activated protein kinase (AMPK) inhibitor). Mechanical withdrawal threshold and thermal withdrawal latency were measured. Necroptosis-associated markers (RIP3 and mixed lineage kinase domain-like protein (MLKL)), autophagy markers (microtubule-associated protein 1A/1B light chain 3B and sequestosome 1), AMPK signaling molecules, and proinflammatory cytokines (interleukin-1β, interleukin-6, and tumor necrosis factor-α) were assessed by Western blotting and immunofluorescence staining. Necrotic cells were detected by propidium iodide (PI) staining.

**Results:**

NP transplantation induced persistent pain hypersensitivity, decreased AMPK phosphorylation, impaired autophagic function, upregulated RIP3 and MLKL expression, increased the number of PI-positive cells, and elevated pro-inflammatory cytokine levels in the lumbar spinal cord of LRP rat models. Intrathecal administration of PDX dose-dependently attenuated pain hypersensitivity, restored AMPK phosphorylation and autophagic function, and suppressed spinal RIP3/MLKL-associated necroinflammation. These beneficial effects were significantly reversed by pretreatment with either CQ or Compound C.

**Conclusion:**

PDX alleviates LRP by restoring AMPK-related autophagic function, thereby suppressing RIP3/MLKL-associated necroinflammation. These findings support PDX as a potential therapeutic candidate for experimental LRP and provide mechanistic insight into the role of SPMs in experimental pain regulation.

## 1 Introduction

Lumbar radicular pain (LRP) is a very common type of neuropathic pain, presenting with symptoms of radicular pain in the distribution of the lumbar dermatomes (Peene et al., 2024). Compared with non-specific low back pain, LRP patients tend to experience more severe pain, disability, healthcare utilization, and cost, thus exerting a heavy socioeconomic impact worldwide (Hasoon et al., 2025). There is increasing evidence showing that the main cause of LRP is the extrusion of nucleus pulposus (NP) tissue, whose occurrence has shown an increasing trend with the increasing aging population all around the world (Soar et al., 2022). The old concept that mechanical compression is the principal cause of LRP has been updated. Modern researches from the fields of both clinical study and translational medicine have revealed that excessive inflammatory reaction induced by NP tissue plays a crucial role in the development of LRP (Cheng et al., 2025). Thus, classical anti-inflammatory approaches (Smith et al., 2020), which work by inhibiting the inflammation induction, have been proved to be effective in LRP (Armon et al., 2025). However, this approach cannot be extensively used because of possible adverse reactions resulting from the compromise of host defense mechanisms (Honvo et al., 2025).

Against this background, the active resolution of inflammation has emerged as a promising alternative (Peh and Chen, 2025). This tightly regulated process is driven by specialized pro-resolving lipid mediators (SPMs), which counter-regulate inflammation and promote tissue repair without impairing host defenses (Anghelache et al., 2025). Conversely, delayed resolution exacerbates autoinflammatory disorders (Panigrahy et al., 2021) and promotes the development of autoimmune diseases (Vomero et al., 2025). Our recent work demonstrated that protectin DX (PDX), a novel member of the SPM family, exerts potent pro-resolving and analgesic effects in LRP. However, the molecular mechanisms through which PDX promotes inflammatory resolution remain incompletely understood.

Necroptosis, which is induced by receptor-interacting protein kinase 3 (RIP3) and mixed lineage kinase domain-like protein (MLKL), has been found to have a connection with the dysregulation of the inflammatory process (Wu et al., 2021). Numerous studies have shown that the cells that undergo RIP3/MLKL-associated necroptotic signaling initiate the immune response along with the inflammation due to the production of damage-associated molecular patterns (DAMPs) (Zhou et al., 2024). The release of DAMPs arising from this process further increases necrotic cells and exacerbates inflammation (Nair et al., 2025), creating a vicious cycle referred to as necroinflammation (Linkermann, 2019). The role of RIP3/MLKL-associated necroptotic signaling and necroinflammation in the development of certain neuropathic diseases has been reported in recent studies (Xu et al., 2024). However, the causative role of RIP3/MLKL-associated necroinflammation in inflammatory pathogenesis of LRP is not yet established. In addition, necroptosis may contribute to the persistence of inflammation by blocking the signaling of SPMs (Gerlach et al., 2020; Zhao et al., 2021), thus perpetuating the necroptosis-necroinflammation pathway in a positive feedback fashion. Conversely, SPMs enhance cellular metabolism and facilitate the efficient clearance of necrotic debris, thereby interrupting this pathological cycle (Fredman and Khan, 2023). These findings raise the possibility that PDX may exert its pro-resolving effects by suppressing the overactive RIP3/MLKL-associated necroinflammation.

Autophagy, a fundamental self-regulatory process that maintains cellular homeostasis, also modulates inflammatory signaling. Emerging evidence indicates that crosstalk between necroptosis and autophagy coordinately regulates cell fate and inflammatory status (Liu et al., 2023; Liu et al., 2018). Adenosine monophosphate-activated protein kinase (AMPK), a ubiquitously expressed protein kinase, promotes autophagic progression through multiple regulatory pathways (Feng et al., 2024). Recent studies have also identified PDX as a novel AMPK activator (White et al., 2014; Piao et al., 2020). These observations raise the question of whether PDX exerts its therapeutic effects in LRP by modulating the AMPK-autophagy-necroinflammation pathway.

To address these knowledge gaps, we first investigated whether the RIP3/MLKL-associated necroinflammation contributes to the progression of LRP induced by non-compressive NP herniation. We then investigated whether the analgesic and pro-resolving effects of PDX are associated with AMPK activation and subsequent modulation of autophagy, leading to suppression of the downstream RIP3/MLKL-associated necroinflammation.

## 2 Materials and methods

### 2.1 Experimental animals

Adult male Sprague-Dawley rats weighing 250–350  g were purchased from Beijing Vital River Laboratory Animal Technology (Beijing, China). The rats were housed individually under standard specific-pathogen-free conditions at an ambient temperature of 23 °C ± 1 °C and on a 12-h light/dark cycle. Standard rodent chow and sterile water were provided ad libitum throughout the experimental period. All animal protocols were approved by the institutional animal care and use committee. All procedures were performed in accordance with the National Institutes of Health Guide for the Care and Use of Laboratory Animals, with every effort made to minimize animal suffering. Rats were randomly assigned to experimental groups using a random number table. Sample sizes were determined based on previous studies using similar experimental models and our preliminary experiments.

### 2.2 Establishment of the LRP rat model

Rats were anesthetized with sodium pentobarbital (40 mg/kg, i.p.). After blunt separation of the paravertebral muscles, the L5-L6 vertebral lamina was carefully removed to expose the epidural space. A sterile PE-10 catheter was then gently inserted into the subarachnoid space. Successful intrathecal catheterization was confirmed by the spontaneous outflow of cerebrospinal fluid (CSF). The external end of the catheter was tunneled subcutaneously to the cranial region and secured with sterile medical adhesive tape. All rats were closely monitored for three consecutive days after catheter implantation. Only rats without apparent neurological deficits, including hindlimb paresis, abnormal gait, or urinary or fecal incontinence, were included in subsequent experiments.

The non-compressive LRP model was established as previously described (Hong et al., 2024). Under sodium pentobarbital anesthesia (40 mg/kg, i.p.), the right L5 DRG was surgically exposed. Autologous NP tissue was harvested from two caudal intervertebral discs of the same rat and microsurgically transplanted onto the surface of the L5 DRG. Care was taken to avoid mechanical compression. Rats in the sham-operated group underwent the same surgical procedure, including DRG exposure, but without NP transplantation.

### 2.3 Intrathecal drug administration

Drugs were administered intrathecally through indwelling PE-10 catheters (Braintree Scientific, USA). All drugs were freshly prepared on the day of administration. PDX (Cayman Chemical, USA) was reconstituted according to the supplier’s instructions. Briefly, the solvent was evaporated under a gentle stream of nitrogen, and the residue was dissolved in sterile phosphate-buffered saline (PBS) to final concentrations of 1, 10, and 20 ng/μL, corresponding to doses of 10, 100, and 200 ng/10 μL, respectively. GSK'872 (a selective RIP3 inhibitor; Selleck, USA), chloroquine (CQ; an autophagy inhibitor; Sigma, USA), and Compound C (an AMPK inhibitor; Sigma, USA) were initially dissolved in dimethyl sulfoxide (DMSO) to prepare stock solutions and subsequently diluted with sterile PBS to the required working concentrations. The final DMSO concentration in all injectates was ≤0.1%.

The intrathecal doses were 11 μg/10 μL for GSK'872 (Xue et al., 2022), 0.1 μg/10 μL for CQ (Jin et al., 2018), 5 μg/10 μL for Compound C (Ling et al., 2017), and 10, 100, or 200 ng/10 μL for PDX. The vehicle consisted of sterile PBS containing 0.1% DMSO. Each intrathecal injection was administered in a volume of 10 μL through the indwelling PE-10 catheter, followed by a 10-μL flush of sterile saline to ensure complete delivery. The experimental groups received the designated compound or vehicle once daily during the first three postoperative days. The sham-operated and LRP model groups received no intrathecal intervention throughout the experimental period.

### 2.4 Assessment of pain-related behaviors

Before behavioral testing, each rat was allowed to acclimate to the testing environment for 30 min to minimize environmental stress. As previously described (Deuis et al., 2017), the mechanical withdrawal threshold (MWT) was assessed using von Frey filaments (Stoelting, USA) according to the up-down method. Thermal withdrawal latency (TWL) was measured using a BME-410A thermal dolorimeter (Institute of Biomedical Engineering, China) set at 50% intensity, with a cutoff time of 20 s. All behavioral assessments were performed by investigators blinded to group allocation.

### 2.5 Western blot analysis

Rats were deeply anesthetized and euthanized by aortic exsanguination. Ipsilateral L4-L6 spinal dorsal horn (SDH) tissues were rapidly collected on ice. For protein extraction, the tissues were homogenized in RIPA lysis buffer (Boster, China) according to the manufacturer’s instructions. Equal amounts of protein (40 μg per sample) were separated by 8%–15% gradient SDS-PAGE and electrophoretically transferred onto polyvinylidene difluoride membranes (Millipore, Billerica, USA). The membranes were blocked with 5% nonfat dry milk for 1 h at room temperature and then incubated overnight at 4 °C with the following primary antibodies: mouse anti-GAPDH (sc-32233; 1:500; Santa, USA), mouse anti-RIP3 (sc-374639; 1:500; Santa, USA), rabbit anti-MLKL (DF7412; 1:1,000; Affinity, USA), rabbit anti-IL-1β (AF5103; 1:1,000; Affinity, USA), rabbit anti-IL-6 (DF6087; 1:1,000; Affinity, USA), rabbit anti-TNF-α (17590-1-AP; 1:2,000; Proteintech, China), rabbit anti-LC3 (14600-1-AP; 1:1,000; Proteintech, China), rabbit anti-p62 (A19700; 1:500; ABclonal, China), rabbit anti-p-AMPK (2535; 1:1,000; CST, USA), and rabbit anti-AMPK (5832; 1:1,000; CST, USA). After washing with Tris-buffered saline containing Tween 20, the membranes were incubated for 1 h at room temperature with horseradish peroxidase-conjugated goat anti-rabbit IgG (BA1054; 1:5,000; Boster, China) or goat anti-mouse IgG (BA1050; 1:5,000; Boster, China). Protein bands were visualized using an enhanced chemiluminescence substrate (Millipore, USA) and imaged with a GenoSens 2000 Gel Imaging System (Clinx, China). Band intensities were quantified using Image-Pro Plus software and normalized to GAPDH expression.

### 2.6 Detection of dying cells

On postoperative day 7, propidium iodide (PI; 10 mg/kg; Servicebio, China) was administered intraperitoneally 60 min before tissue collection. Following deep anesthesia, the rats were euthanized by aortic exsanguination, and the L4-L6 spinal dorsal horn (SDH) segments were dissected. The tissues were immediately embedded in optimal cutting temperature (OCT) compound and rapidly frozen at −20 °C. Cryosections (10 μm) were prepared, washed with phosphate-buffered saline (PBS), and counterstained with 4′,6-diamidino-2-phenylindole (DAPI; Boster, China) for 5 min at room temperature. Images were acquired using a fluorescence microscope (Olympus, Japan). For PI and MLKL double staining, following PI administration and tissue sectioning, the sections were incubated with an anti-MLKL antibody and then with a DyLight 488-conjugated secondary antibody, as described in Section 2.7. The sections were subsequently counterstained with DAPI.

### 2.7 Immunofluorescence

Ipsilateral L4-L6 spinal dorsal horn tissues were embedded in OCT compound and rapidly frozen at −20 °C. Cryosections (10 μm) were prepared, thoroughly rinsed with sterile PBS, and blocked with QuickBlock™ Buffer (Beyotime, China) for 10 min at room temperature. The sections were then incubated overnight at 4 °C with a primary antibody cocktail containing rabbit anti-MLKL (DF7412; 1:150; Affinity, USA), mouse anti-GFAP (GB12096; 1:500; Servicebio, China), mouse anti-Iba1 (GB12105; 1:500; Servicebio, China), and mouse anti-NeuN (66836-1-Ig; 1:100; Proteintech, USA). After washing with PBS, the sections were incubated in the dark at 37 °C for 60 min with DyLight 488-conjugated goat anti-rabbit IgG (1:500; Abbkine, USA) and DyLight 594-conjugated goat anti-mouse IgG (1:500; Abbkine, USA). The sections were then counterstained with DAPI (Boster, China) for 5 min at room temperature, washed with PBS, and mounted using an antifade mounting medium. Fluorescence images were acquired using a fluorescence microscope (Olympus, Japan).

### 2.8 Statistical analysis

All behavioral assessments, Western blot quantification, and immunofluorescence analysis were performed by investigators blinded to group allocation. For all experiments, n denotes the number of independent animals per group. All quantitative data were presented as the mean ± standard error of the mean (SEM). Statistical analyses were performed using IBM SPSS Statistics 21.0 (IBM, USA), and graphs were generated using GraphPad Prism 8.02 (GraphPad Software, USA). All variables followed a Gaussian distribution except MWT, which was log10-transformed before analysis. Behavioral data were analyzed using two-way repeated-measures ANOVA with treatment and time as factors, including evaluation of the treatment × time interaction, followed by Tukey’s multiple-comparisons test. Biochemical data obtained by Western blotting and immunofluorescence were analyzed using one-way ANOVA followed by Tukey’s multiple-comparisons test. Statistical significance was defined as *P < 0.05*.

## 3 Results

### 3.1 Temporal expression and cellular localization of necroptosis-associated markers in the lumbar spinal cord of LRP rats

To investigate the contribution of the RIP3/MLKL-associated necroinflammation to LRP, we examined the temporal expression of the necroptosis-associated markers RIP3 and MLKL in the lumbar spinal cord of LRP rats (Figures 1A–C). Quantitative immunoblotting showed that RIP3 and MLKL protein levels increased in a time-dependent manner, peaking on postoperative days 3 and 7 before declining by day 14 (*P* < 0.05 vs. sham). The temporal expression patterns of RIP3 and MLKL closely paralleled the progression of pain hypersensitivity in LRP rats (Figures 2A,B). This temporal association suggests that necroptosis-related signaling is closely linked to the onset and progression of LRP.

**FIGURE 1:**
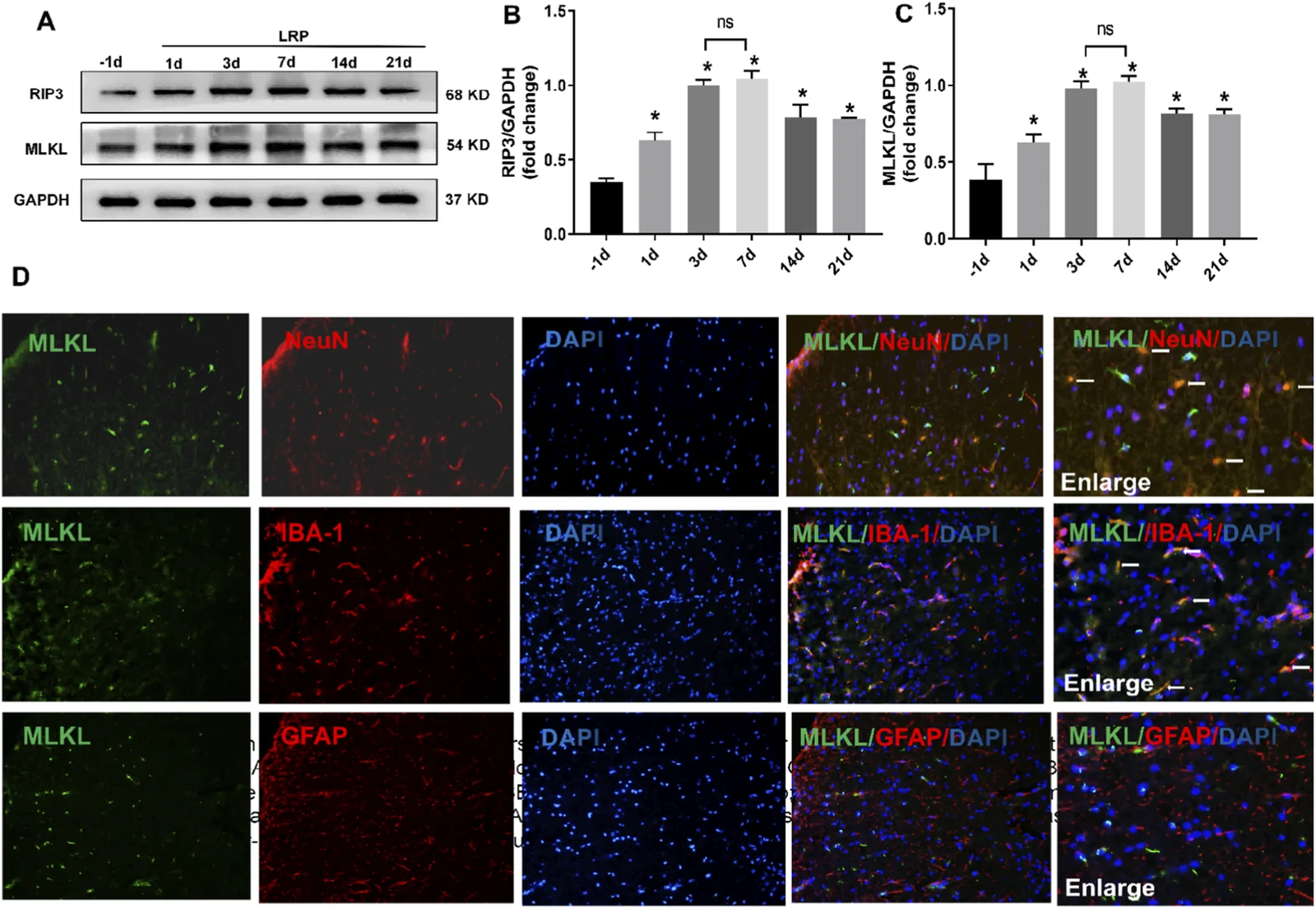
Necroptotic signaling activation in the lumber spinal dorsal horn of LRP model rats. **(A)** Representative Western blot bands showing the time-dependent expression changes of necroptotic signaling markers (IP3 and MLKL) in the lumbar spinal cord of LRP model rats (n = 3 rats / group). **(B, C)** Quantitative analysis of relative protein levels of RIPS3 **(B)** and MLKL **(C)**. Data are shown as mean ± SEM. Statistical comparisons among multiple groups were conducted by one-way ANOVA followed by Tukey’s post hoc test (*p* < 0.05 vs. sham group). **(D)** Immunofluorescence staining showing the cellular localization of MLKL (green) with the neuronal marker NeuN, microglial marker lba-1, and astrocytic marker GFAP, glial fibrillary acidic protein; lba-1, ionized calcium-binding adapter molecule 1; MLKL, mixed lineage kinase domain-like protein; NeuN, neuronal nuclear antigen; RIP3, receptor-interacting serine/threonine-protein kinase 3; LRP, lumbar radicular pain.

**FIGURE 2:**
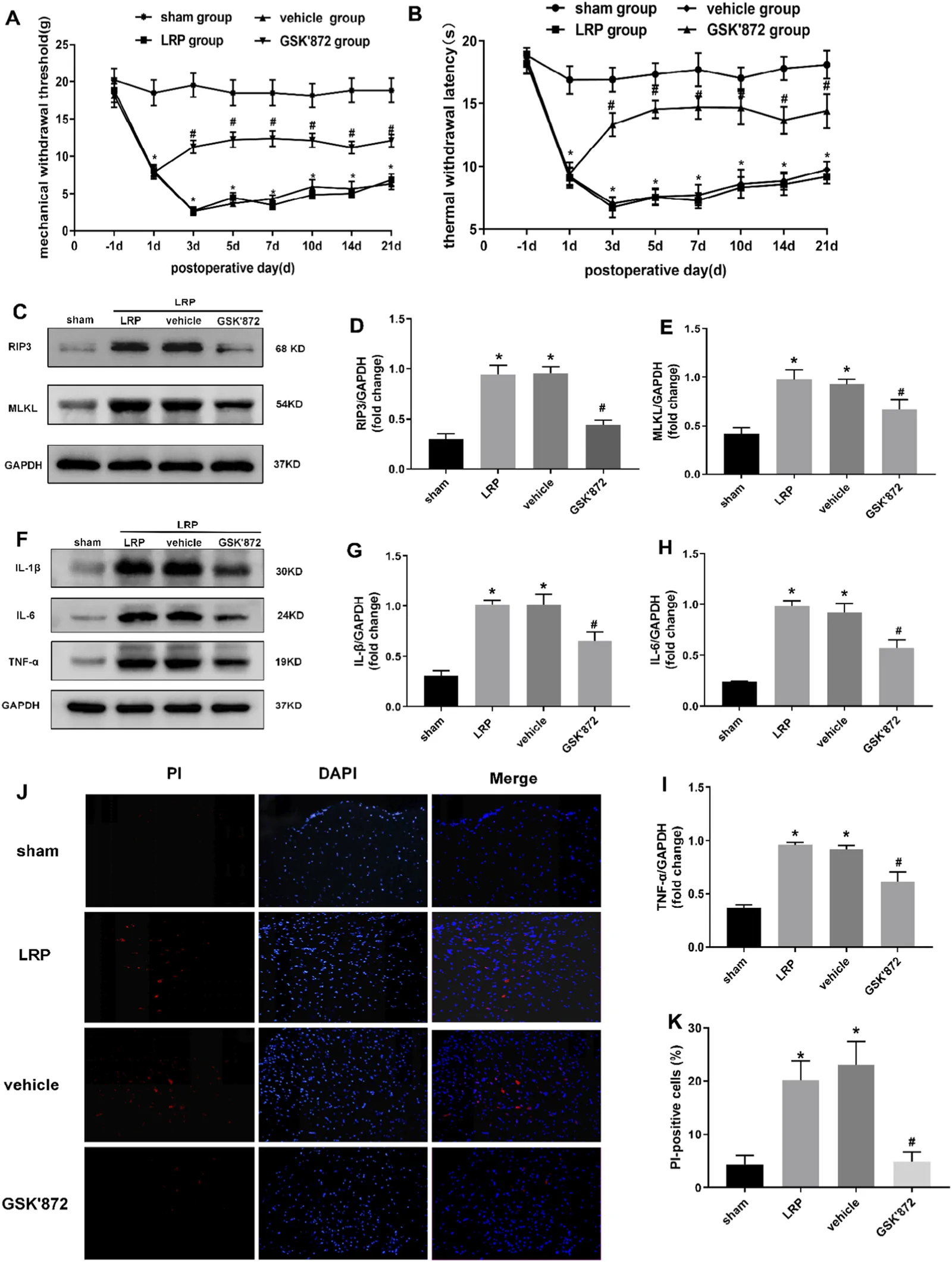
Effects of GSK’872 on pain behaviours and spinal neuroinflammation in LRP rats. **(A, B)** GSK’872 significantly improved MWT and TWL in LRP rats (n = 9 rats/group). Data are presented as mean ± SEM. Two-way repeated-measures ANOVA was performed, followed by Tukey’s post hoc test (*p < 0.05 vs. sham group; #*p* < 0.05 vs. vehicle group). **(C)** Representative western blots showing the protein expression of RIP3 and MLKL (n = 4 rats/group). **(F)** Representative Western blots showing protein expression of IL-1β, IL-6, AND TNF-α (n = 3 rats/group). **(J)** Representative images of PI staining for necrotic cells (red) (n = 3 rats/group); scale bars: 100 µm. **(D, E, G-I, K)** Quantitative analyses of protein levels of RIP3 **(D)**, MLKL **(E)**, IL-1β **(G)**, IL-6 **(H)**, and TNF-α **(I)**, as well as PI-positive cell number **(K)**. Data are presented as mean ± SEM. Statistical comparisons were performed using one-way ANOVA followed by Tukey’s post hoc test (*p < 0.05 vs. sham group; #*p* < 0.05 vs. vehicle group). Abbreviations: IL-1β, interleukin-1β; IL-6, interleukin-6; LRP, lumbar radicular pain; MLKL, mixed lineage kinase domain-like protein; MWT, mechanical withdrawal threshold; PI, propidium iodide; RIP3, receptor-interacting protein kinase 3; DAPI, 4’,6-Diamidino-2-phenylindole; TNF-α, tumor necrosis factor-α; TWL, thermal withdrawal latency.

To determine the cellular localization of necroptosis-related signaling in the spinal cord of LRP rats, immunofluorescence colocalization assays were performed on spinal cord sections collected 7 days after model induction, when necroptosis-related marker expression and pain hypersensitivity peaked. MLKL was co-immunostained with the following cell-type-specific markers: neuronal nuclei (NeuN) for neurons, ionized calcium-binding adapter molecule 1 (Iba1) for microglia, and glial fibrillary acidic protein (GFAP) for astrocytes. MLKL predominantly colocalized with NeuN-positive neurons and Iba1-positive microglia, whereas colocalization with GFAP-positive astrocytes was not detected (Figure 1D). These findings indicate that neurons and microglia are the principal cell types undergoing necroptosis-related signals in the spinal cord of LRP rats.

Given the close temporal association between necroptosis-related signaling and pain development, we next examined whether pharmacological inhibition of necroptosis-related signaling could attenuate LRP pathology.

### 3.2 Pharmacological inhibition of RIP3 attenuates spinal necroinflammation and pain hypersensitivity in LRP rats

Given the central role of RIP3 in programmed necroptosis and the subsequent inflammatory cascade, GSK'872, a selective RIP3 inhibitor, was administered intrathecally after LRP induction. As shown in Figures 2A,B, baseline 50% MWT and TWL did not differ significantly among the groups before model establishment (*P* > 0.05). Following NP transplantation onto the DRG, the 50% MWT and TWL were markedly reduced in the model, vehicle, and GSK'872 groups compared with the sham group (*P* < 0.05), indicating the successful induction of mechanical allodynia and thermal hyperalgesia. No significant differences in the 50% MWT or TWL were observed between the model and vehicle groups (*P* > 0.05). Intrathecal GSK'872 administration significantly increased the 50% MWT and prolonged the TWL in LRP rats (*P* < 0.05 vs. vehicle). These findings demonstrate that pharmacological inhibition of RIP3 with GSK'872 alleviates ipsilateral neuropathic pain behaviors in LRP rats.

The inhibitory effect of GSK'872 on necroptosis-related signaling was further evaluated at the molecular level. As shown in Figures 2C–E, RIP3 and MLKL protein levels were markedly elevated in both the LRP model and vehicle groups compared with the sham group (*P* < 0.05), with no significant difference between the model and vehicle groups (*P* > 0.05). These findings indicate that vehicle administration did not alter necroptosis-related signaling. In contrast, GSK'872 administration significantly reduced RIP3 and MLKL expression (*P* < 0.05 vs. vehicle), confirming effective inhibition of the RIP3-MLKL pathway. These findings support the involvement of RIP3/MLKL-associated signaling in LRP rats.

To examine the relationship between RIP3/MLKL-associated signaling and necroinflammation, the expression levels of the proinflammatory cytokines tumor necrosis factor-α (TNF-α), interleukin-1β (IL-1β), and IL-6 were quantified (Figures 2F–I). All three cytokines were significantly upregulated in LRP rats (*P* < 0.05 vs. sham). GSK'872-mediated RIP3 inhibition significantly attenuated the upregulation of these necroinflammatory mediators (*P* < 0.05 vs. vehicle). PI staining further showed that the density of necrotic cells in the spinal cord was significantly higher in LRP rats than in sham controls (Figures 2J,K; *P* < 0.05). Vehicle treatment had no effect on necrotic cell accumulation (*P* > 0.05 vs. LRP model), whereas GSK'872 markedly suppressed the progression of necrotic cells (*P* < 0.05 vs. vehicle). Collectively, these findings support the involvement of RIP3/MLKL-associated necroinflammation in pain hypersensitivity in experimental LRP.

### 3.3 PDX suppresses the RIP3/MLKL-associated necroinflammation and alleviates LRP in a dose-dependent manner

We next evaluated the therapeutic effects of PDX in LRP rats. As shown in Figures 3A,B, baseline 50% MWT and TWL measured 1 day before surgery (day −1) did not differ significantly among the groups (*P* > 0.05). By postoperative day (POD) 1, both the 50% MWT and TWL had decreased significantly in all intervention groups compared with the sham group (*P* < 0.05). During PODs 2–7, the LRP group continued to exhibit significantly lower 50% MWT and TWL values than the sham group (*P* < 0.05). No significant differences were observed between the LRP and vehicle-treated groups (*P* > 0.05). Intrathecal administration of PDX at doses of 10, 100, or 200 ng produced dose-dependent increases in the 50% MWT and TWL (*P* < 0.05 vs. vehicle). Further quantitative analysis showed a significant dose-dependent increase in TWL between the 10- and 100-ng PDX groups (*P* < 0.05). Collectively, these findings demonstrate that intrathecal PDX administration attenuates ipsilateral mechanical allodynia and thermal hyperalgesia in LRP rats.

**FIGURE 3:**
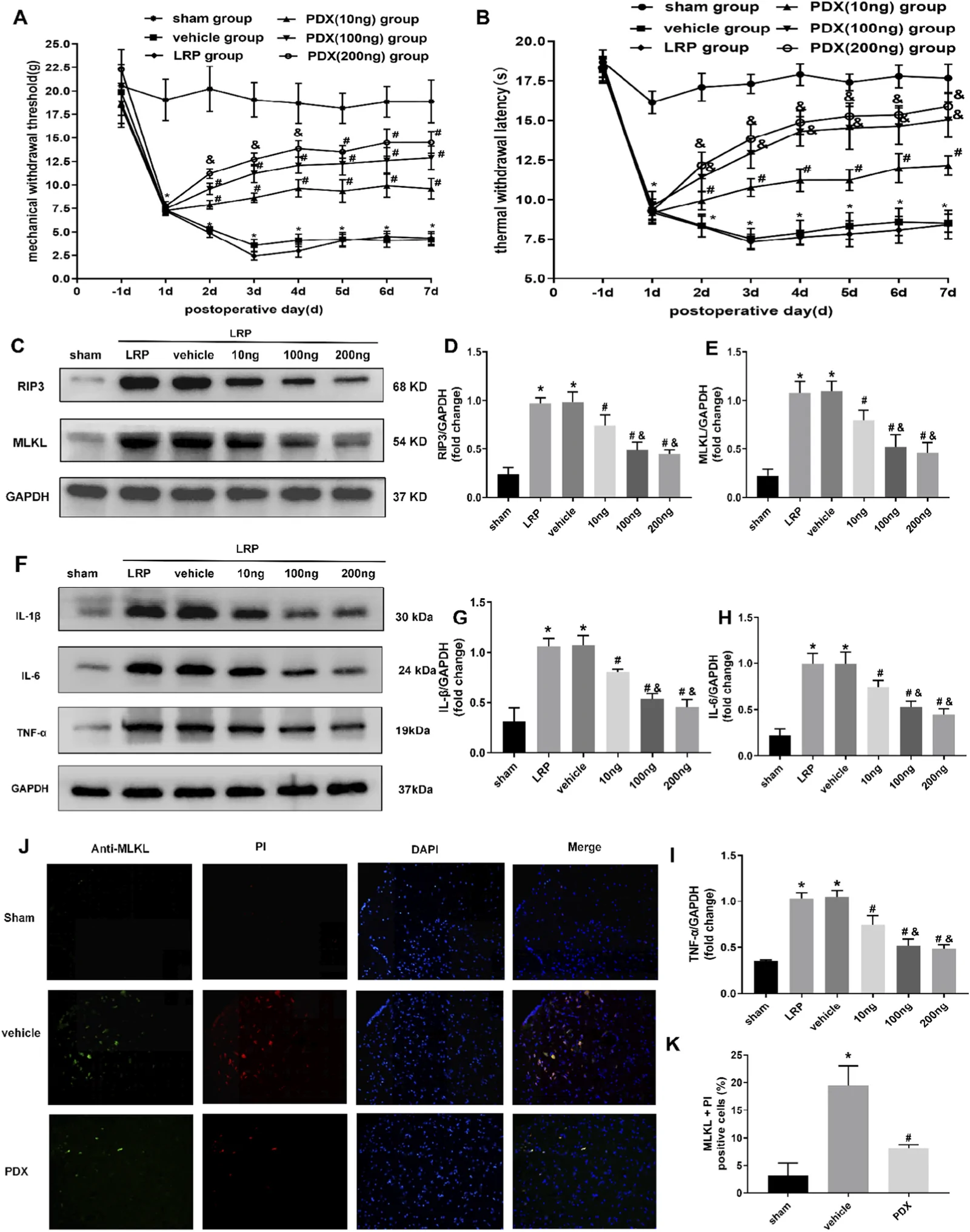
Effects of Protectin DX on pain behaviors and spinal necroinflammation in LRP rats. **(A, B)** PDX (10ng, 100ng or 200ng) treatment prominently meliorated the 50% MWT and prolonged TWL of LRP rat models (n = 9 rats/group). Data are presented as the mean ± SEM; Two-way ANOVA was used for comparisons among groups followed by Turkey’s multiple comparisons test (*P < 0.05 versus sham group; #P < 0.05 versus vehicle group; & P < 0.05 versus 10ng group). **(C, F)** Representative western blots showing protein expression of RIP3, MLKL, IL-1β, IL-6, and TNF-α (n = 4 rats/group). **(J)** Double immunofluorescence staining for MLKL (green) and PI (red) in the lumbar spinal cord on postoperative day 7 ((n = 3 rats/group). scale bars: 100µm. **(D, E, G-I, K)** Quantitative analyses of protein levels of RIP3 **(D)**, MLKL **(E)**, IL-1β **(G)**, IL-6 **(H)**, and TNF-α **(I)**, as well as MLKL/PI double-positive cell numbers **(K)**. Data are presented as mean ± SEM. Statistical comparisons were performed using one-way ANOVA followed by Tukey’s post hoc test (*p < 0.05 versus sham group; #*p* < 0.05 versus vehicle group; & *p* < 0.05 versus 10ng group). Abbreviations: IL-1β, interleukin-1β; IL-6, interleukin-6; LRP, lumbar radicular pain; MLKL, mixed lineage kinase domain-like protein; MWT, mechanical withdrawal threshold; PI, propidium iodide; RIP3, receptor-intracting protein kinase 3; DAPI, 4’ 6-Diamidino-2-phenylindole; TNF-α, tumor necrosis factor-α; TWL, thermal withdrawal latency; Protectin DX, PDX.

To determine whether PDX exerts its antinociceptive effects by targeting the RIP3/MLKL-associated necroinflammation, we administered intrathecal PDX at doses of 10, 100, or 200 ng and examined key molecules involved in RIP3/MLKL-associated necroinflammation. Western blotting showed that RIP3 and MLKL protein levels were significantly elevated in the lumbar spinal cord of LRP rats 7 days after model induction (Figures 3C–E; *P* < 0.05 vs. sham), with no significant difference between the LRP model and vehicle groups (*P* > 0.05). Intrathecal PDX treatment dose-dependently reduced spinal RIP3 and MLKL expression (*P* < 0.05 vs. vehicle), with significantly greater inhibition in the 100-ng group than in the 10-ng group (*P* < 0.05). Necrotic cells were subsequently assessed by immunofluorescence double labeling for MLKL and PI. As shown in Figures 3J,K, the number of MLKL/PI double-positive cells was markedly increased in the vehicle group compared with the sham group (*P* < 0.05). Intrathecal administration of 100 ng PDX significantly reduced the density of MLKL/PI double-positive cells in the lumbar spinal cord (*P* < 0.05 vs. vehicle), indicating that PDX suppresses spinal MLKL/PI double-positive cells in LRP rats. We further evaluated the effects of PDX on inflammatory cytokine expression (Figures 3F–I). Compared with sham-operated controls, the LRP model group exhibited significantly higher levels of the proinflammatory cytokines IL-1β, IL-6, and TNF-α (*P* < 0.05), with no significant differences between the LRP and vehicle-treated groups (*P* > 0.05). Intrathecal PDX at doses of 10, 100, or 200 ng dose-dependently reduced IL-1β, IL-6, and TNF-α expression (*P* < 0.05 vs. vehicle). Consistent with the preceding findings, the anti-inflammatory effect was significantly greater in the 100-ng PDX group than in the 10-ng group (*P* < 0.05).

Collectively, these results suggest that PDX, similar to the selective RIP3 inhibitor GSK'872, inhibits RIP3/MLKL-associated necroinflammation, thereby contributing to its therapeutic effects against LRP.

### 3.4 PDX promotes autophagic function through AMPK signaling in the spinal cord of LRP rats

To investigate the upstream mechanism, we assessed AMPK phosphorylation and the autophagy markers LC3B and p62 in the spinal cord of LRP rats (Figure 4). Compared with the sham group, vehicle-treated LRP rats exhibited a significant decrease in the spinal p-AMPK/AMPK ratio (*P* < 0.05), accompanied by increased LC3B and p62 expression (*P* < 0.05). This molecular profile was consistent with AMPK inactivation and impaired autophagic function. Intrathecal PDX administration dose-dependently reversed the reduction in the p-AMPK/AMPK ratio (*P* < 0*.05* vs. vehicle; *P* < 0.05 vs. 10 ng PDX) and restored autophagic function, as indicated by increased LC3B expression and reduced p62 accumulation in spinal cord tissue (Figures 4A–E; *P* < 0.05 vs. vehicle; *P* < 0.05 vs. 10 ng PDX).

**FIGURE 4:**
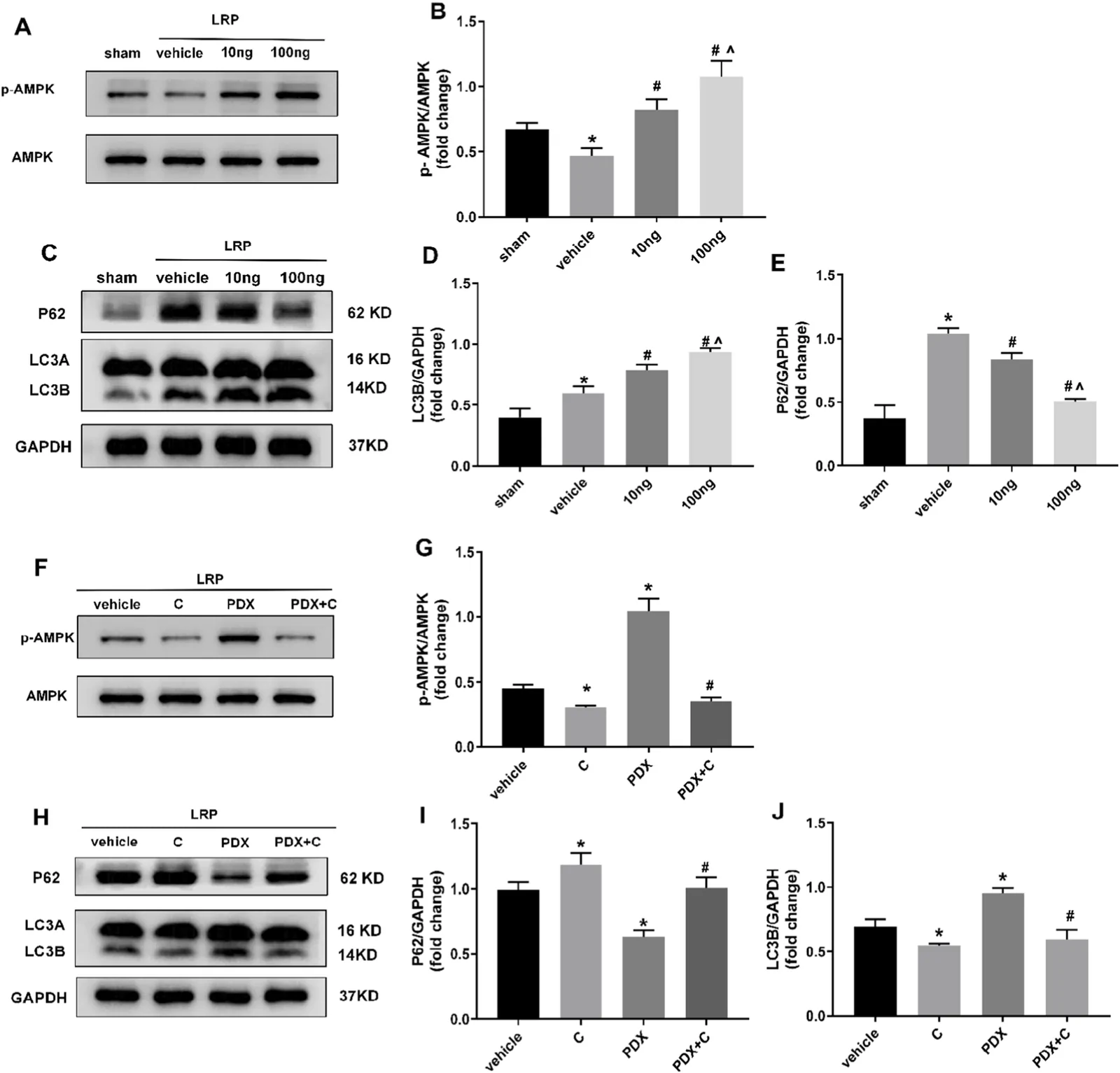
PDX facilities autophagic function in an AMPK-dependent manner in lumbar spinal cord tissue of LRP rat models **(A, C)** Representative western blots showing p-AMPK, AMPK, LC3B, and p62 protein levels in the lumbar spinal cord following administration of different doses of PDX (n = 3 rats/group). **(B, D, E)** Quantitative analyses of p-AMPK/AMPK ratio **(B)** and LC3B **(D)** and p62 **(E)** protein levels. Data are presented as mean ± SEM. One-way ANOVA was performed, followed by Tukey’s post hoc test (*p < 0.05 vs. sham group; #*p* < 0.05 vs.vehicle group, ^ *p* < 0.05 vs. 10 ng PDX group). **(F,H)** Representative western blots showing p-AMPK, AMPK, LC3B, and p62 protein levels in the lumbar spinal cord following PDX administration with or without Compound C pretreatment (n = 3 rats/group). **(G, I, J)** Quantitative analyses of p-AMPK/AMPK ratio **(G)**, p62 **(I)** and LC3B **(J)** protein levles. Data are presented as mean ± SEM. One-way ANOVA was performed, followed by Tukey’s post hoc test (*p* < 0.05 vs.vehicle group; #*p* < 0.05 vs. PDX monotherapy grouop). Abbreviations: AMPK, adenosine monophosphate-activated protein kinase; C: Compound C; LC3B, microtubule-associated protein 1A/1B light chain 3B; LRP, lumbar radicular pain; PDX, protectin DX; p-AMPK, phosphorylated AMPK; p62, sequestosome 1.

To determine whether AMPK is required for the PDX-mediated restoration of autophagic function, Compound C, a commonly used AMPK inhibitor, was administered. As shown in Figures 4F,G, intrathecal Compound C significantly reduced the spinal p-AMPK/AMPK ratio in LRP rats (*P* < 0.05 vs. vehicle), confirming effective inhibition of AMPK activity. Consistent with the preceding results, PDX monotherapy (100 ng) markedly increased LC3B and decreased p62 expression (*P* < 0.05 vs. vehicle). However, pretreatment with Compound C significantly reversed these effects of PDX on autophagic function (*P* < 0.05 vs. PDX monotherapy). These pharmacological reversal findings support the involvement of AMPK signaling in the PDX-associated restoration of spinal autophagic function in LRP rats.

### 3.5 Autophagy contributes to PDX-mediated suppression of necroinflammation

To determine whether autophagy mediates the effects of PDX on necroinflammation, CQ, an autophagy inhibitor, was used for pharmacological intervention. As shown in Figures 5A–C, CQ treatment alone further increased LC3B and p62 levels in LRP rats compared with the vehicle-treated group (*P* < 0.05), confirming effective blockade of autophagic function. CQ also exacerbated the NP-induced upregulation of RIP3, MLKL, and the proinflammatory cytokines IL-1β, IL-6, and TNF-α in the spinal cord (Figures 5D–J; *P* < 0.05 vs. vehicle), indicating that impaired autophagy promotes neuroinflammation.

**FIGURE 5:**
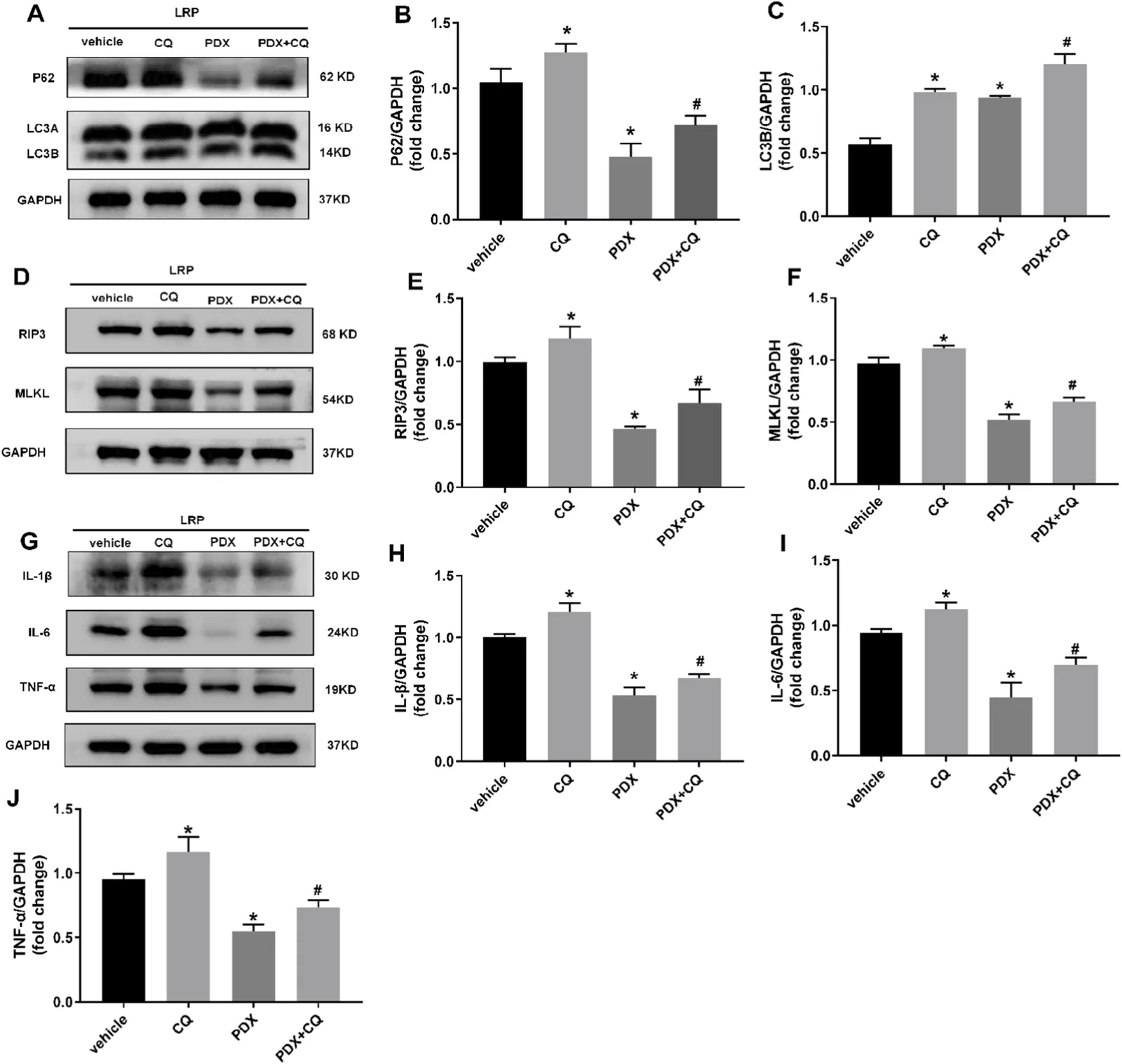
Autophagic function blockade reverses the inhibitory effect of PDX on necroinflammation-related proteins in lumbar spinal cord tissue of LRP rat models **(A, D, G).** Representative western blots showing protein levels of LC3B, p62, RIP3, MLKL,IL-1β, IL-6, and TNF-α in the lumbar spinal cord following PDX administration with or without CQ pretreatment (n = 3 rats/group). **(B, C, E, F, H-J)** Quantitative analyses of the changes in protein levels of LC3B **(B)**, p62 **(C)**, RIP3 **(E)**, MLKL **(F)**, IL-1β **(H)**, IL- **(I)** and TNF-α **(J)**. CQ, significantly reversed the improving autophagy function and reducing necroinflammation of intrathecal administration of PDX. Values are displayed as mean ± SEM, One-way ANOVA was used for comparisons among groups followed by Turkey’s multiple comparisons test (*p < 0.05 versus vehicle group; #*p* < 0.05 versus PDX group). RIP3: receptor-interacting protein 3; mlkl: mixed lineage kinase domain-like protein; IL-β: Interleukin-1 beta; IL-6: interleukin-6; TNF-α: TUMOUR NECROSIS FACTO-ALPHA;PDX, protectin DX; CQ: Chloroquine; LC3B, microtubule-associated protein 1A/1B light chain 3B; LRP: Lumbar radicular pain: p62, sequestosome 1.

Consistent with the preceding findings, PDX monotherapy (100 ng) significantly increased LC3B expression, decreased p62 expression, and reduced the spinal protein levels of RIP3, MLKL, IL-1β, IL-6, and TNF-α (*P* < 0.05 vs. vehicle; Figure 5). However, CQ pretreatment significantly reversed the PDX-mediated suppression of necroinflammation-related proteins (*P* < 0.05 vs. PDX monotherapy; Figure 5). These findings suggest that autophagy contributes to the anti-necroinflammatory effects of PDX.

## 4 Discussion

In this study, we established a rat model of LRP induced by non-compressive NP herniation. This model minimized confounding by mechanical compression and more closely reproduced the inflammatory component of clinical LRP caused by NP exposure. Our principal findings showed that intrathecal PDX, a novel SPM, dose-dependently restored AMPK-mediated autophagy, thereby suppressing spinal RIP3/MLKL-associated necroinflammation and ultimately alleviating LRP-related pain behaviors. These findings support PDX as a potential therapeutic candidate for experimental LRP and provide mechanistic insight into the role of SPMs in experimental pain regulation.

One of the major findings in this study is the involvement of RIP3/MLKL-associated necroinflammation in LRP. The RIP3/MLKL is known to play an essential role in the regulation of necroptosis and subsequent inflammation (Zhou et al., 2024). There is an increasing body of evidence on how the activation of the necroptosis-necroinflammation axis through RIP3/MLKL results in increased tissue damage through the influx of pro-inflammatory cells and impaired immune homeostasis (Chen et al., 2018). The process is associated with the development of various types of inflammation, including neuroinflammatory disorders (Xu et al., 2024), diabetes (Pang et al., 2024), and chronic kidney disease (Chen et al., 2018). Intervention at this stage of the cascade can therefore be considered as a potential treatment modality (Jost and Höckendorf, 2019). Our results show that spinal RIP3 and MLKL expression is upregulated over time, which follows the course of hypersensitivity in LRP rats. Immunofluorescence studies show that necroptosis-related signals mainly happens in neurons and microglial cells, which suggests that these cells are the major sites where cell death and inflammatory pathways take place in LRP. The functional involvement of this pathway was further supported by pharmacological inhibition of RIP3 with GSK'872, which reduced RIP3/MLKL-associated signaling and PI-positive cells, suppressed proinflammatory cytokine production, and attenuated pain behaviors. Although previous studies have implicated necroptosis-related signals in neuropathic pain associated with spinal cord injury and diabetic neuralgia, its cellular localization, temporal profile, and independent contribution to non-compressive NP-induced LRP have remained unclear. Our findings address this gap by providing the evidence that RIP3/MLKL-associated necroptotic signaling and necroinflammation may contribute to LRP pathology. These findings extend the current understanding of pathophysiological mechanisms underlying LRP and support further investigation of RIP3/MLKL-associated necroinflammation as a potential intervention-related pathway in experimental LRP.

The interplay between necroptosis and inflammatory resolution provides a mechanistic rationale for PDX treatment. As noted above, necroptosis can directly antagonize regenerative processes by impairing SPM signaling (Zhao et al., 2021), thereby favoring pathological remodeling over functional recovery (Fiani et al., 2021). Conversely, recent studies (Gerlach et al., 2020; Zhao et al., 2021) have shown that SPM production is activated as a compensatory negative-feedback response to excessive inflammatory signaling (Godson et al., 2023). The conserved structures of SPMs contribute to the efficient clearance of pathogens and harmful stimuli while promoting tissue repair and regeneration (Ghodsi et al., 2024). PDX may therefore exert its pro-resolving effects by modulating necroptosis-related signals. Consistent with this hypothesis, intrathecal PDX significantly reduced RIP3 and MLKL expression in the lumbar spinal cord of LRP rats. These changes were accompanied by fewer MLKL/PI-positive cells, lower proinflammatory cytokine levels, and significant improvements in mechanical and thermal pain thresholds. These observations indicate that PDX exerts analgesic and anti-inflammatory effects in LRP by inhibiting the RIP3/MLKL-associated necroinflammation. This mechanism extends the current understanding of PDX-mediated pain regulation and supports further investigation of SPMs for the treatment of LRP.

Another important finding was the identification of the upstream mechanism through which PDX regulates necroinflammation. Emerging evidence indicates that close crosstalk between necroptosis and autophagy coordinately regulates cell fate and inflammatory status (Zhang et al., 2023). Two studies of secondary spinal cord injury showed that impaired autophagy caused rapid accumulation of RIP3 and MLKL, thereby inducing necroptosis and neuroinflammation (Liu et al., 2018; Zhang et al., 2023). Conversely, restoration of autophagic function attenuated necroptosis and inflammation and improved neurological function. In the present study, intrathecal CQ, an inhibitor of autophagy, further increased the aberrantly elevated levels of the necroptosis effectors RIP3 and MLKL and exacerbated the associated inflammatory response (Figures 5D–J). These findings support an association between impaired autophagy and the activation of necroinflammation in LRP. This relationship is also supported by evidence that the autophagic adaptor protein p62 directly interacts with RIP kinases and regulates their stability (Xu et al., 2021) and that genetic ablation of core autophagy genes such as Atg5 modulates necroptosis (Sun et al., 2022).

Having established the autophagy-necroinflammation interaction as a pathological mechanism in LRP, we next investigated the upstream mediator of the therapeutic effects of PDX. Given the established role of AMPK as a major regulator of autophagy and accumulating evidence that PDX activates AMPK, we hypothesized that PDX acts through AMPK-dependent autophagy. Our findings supported this hypothesis in a stepwise manner. First, LRP rats exhibited abnormal AMPK phosphorylation and impaired autophagic function in the spinal cord. Second, intrathecal PDX dose-dependently restored AMPK signaling and autophagic function (Figure 4), and both effects were markedly reduced by AMPK inhibition. These results support an association between AMPK signaling and PDX-induced changes in autophagy-related markers. We subsequently examined whether restoration of autophagic function was required for PDX-mediated suppression of necroinflammation. Consistent with the preceding results, intrathecal PDX restored impaired autophagic function and markedly suppressed the necroinflammation (Figure 5). However, CQ administration before PDX treatment inhibited autophagic function and significantly reversed the PDX-induced reductions in RIP3, MLKL, IL-1β, IL-6, and TNF-α. These findings suggest that PDX suppresses necroinflammation by restoring autophagic function and further define the mechanistic framework underlying the potential use of SPMs in LRP treatment.

Several limitations should be acknowledged. First, phosphorylated MLKL (p-MLKL) was not assessed as a marker of necroptosis. Nevertheless, the increases in total MLKL expression and the number of PI-positive cells support the involvement of necroptosis-associated signaling. Second, the upstream signaling cascade that initiates necroptosis-associated signaling in this model remains unclear. Given the marked increase in TNF-α expression, the TNFR1-RIP1-RIP3 pathway is a plausible candidate. However, direct evidence using approaches such as TNFR1-neutralizing antibodies or RIP1 inhibitors is required. Third, we did not perform live-cell imaging of autophagic dynamics or determine the cellular localization of the autophagic markers LC3B and p62. Our biochemical measurements of LC3B and p62 therefore provide only indirect evidence of autophagy. Future studies using GFP-LC3 transgenic rats or electron microscopy would strengthen these findings. Fourth, the biochemical measurements were performed on whole spinal tissue, cell-type-specific analyses would be required. Fifth, only male rats were used in the present study, because pain responses can be sex-dependent, the generalizability of our findings to female animals remains to be established.

In conclusion, these findings identify the RIP3/MLKL-associated necroptotic signaling and necroinflammation as an important pathological mechanism involved in experimental LRP, and suggest that the analgesic effects and pro-resolving property of PDX may be mediated through modulation of AMPK-related autophagic signaling and its downstream RIP3/MLKL-associated necroinflammation. These findings provide preclinical evidence supporting further investigation of PDX and provide mechanistic insight into the role of SPMs in experimental pain regulation.

## Data Availability

The original contributions presented in the study are included in the article/supplementary material, further inquiries can be directed to the corresponding author.
